# CD147 promotes progression of head and neck squamous cell carcinoma via NF‐kappa B signaling

**DOI:** 10.1111/jcmm.13996

**Published:** 2018-11-12

**Authors:** Binbin Yu, Yan Zhang, Kailiu Wu, Lizhen Wang, Yingying Jiang, Wantao Chen, Ming Yan

**Affiliations:** ^1^ Department of Oral and Maxillofacial‐Head & Neck Oncology Shanghai Ninth People's Hospital College of Stomatology National Clinical Research Center for Oral Diseases Shanghai Key Laboratory of Stomatology & Shanghai Research Institute of Stomatology Shanghai Jiao Tong University School of Medicine Shanghai China; ^2^ Department of Stomatology Xuhui Central Hospital Shanghai China; ^3^ Department of Oral Pathology Shanghai Ninth People's Hospital Shanghai Jiao Tong University School of Medicine Shanghai China

**Keywords:** CD147, head and neck squamous cell carcinoma, NF‐kappa B

## Abstract

CD147/basigin (BSG) is highly upregulated in many types of cancer, our previous study has found that CD147/BSG is highly expressed in head and neck squamous cell carcinoma (HNSCC) stem cells, but its role in HNSCC and the underlying mechanism is still unknown. In this study, we investigated the role of CD147 in the progression of HNSCC. Real‐time PCR, western blot and immunohistochemistry were used to detect the expression of CD147 in total 189 HNSCC tissues in compared with normal tissues. In addition, we used proliferation, colony formation, cell cycle and apoptosis, migration and invasion as well as wound‐healing assay to determine the biological roles of CD147 in HNSCC. Then, a xenograft model was performed to evaluate tumor‐promoting and metastasis‐promoting role of CD147 in HNSCC. The results showed that upregulated CD147 expression was associated with aggressive clinicopathologic features in HNSCC. In addition, CD147 promoted proliferation, migration and reduced the apoptosis phenotype of HNSCC cells in vitro as well as tumor initiation and progression in vivo. Furthermore, we demonstrated that CD147 promoted HNSCC progression through nuclear factor kappa B signaling. Therefore, we concluded that CD147 promoted tumor progression in HNSCC and might be a potential prognostic and treatment biomarker for HNSCC.

## 1 INTRODUCTION

As the seventh most common cancer worldwide, head and neck squamous cell carcinoma (HNSCC) had more than 300 000 new cases and caused over 140 000 deaths worldwide.^1^ In recent years, neck dissection improved overall survival (OS) rate and decreased the relapse rate, but caused injury to the anatomically contiguous and prolonged patients’ hospital stay.^2^ Precise treatment targeting EGFR reduced patients’ recurrence and metastasis, but a subset of tumors remains ineffective.^3^, ^4^ And the mechanisms of carcinogenesis and development of HNSCC are poorly understood. Therefore, it is of great importance to investigate high precision predictive biomarkers of HNSCC for future treatment.

Our previous study has reported that CD147/basigin (BSG) is highly expressed in HNSCC sphere‐forming cells compared to adherent cells,^5^ which suggest that CD147/BSG might be a predictable marker for tumor progression of HNSCC. CD147, a transmembrane glycoprotein, belongs to the immunoglobulin superfamily and is widely expressed on the surface of cancer cells.^6^ Reports showed that CD147 was over‐expressed in many types of cancer, such as human malignant melanoma, hepatocellular carcinoma, lung cancer, gastric cancer, breast cancer and so on.^7^, ^8^, ^9^, ^10^, ^11^, ^12^ CD147 is also reported to involve in tumor progression, angiogenic function and immune response.^13^, ^14^

Furthermore, many studies demonstrated that CD147 could stimulate the activation of nuclear factor kappa B (NF‐kappa B) pathway.^15^, ^16^, ^17^ The NF‐kappa B pathway is reported to elevate cancer cell proliferation, metastasis and reduce cancer cell apoptosis.^11^, ^18^ And the family of NF‐kappa B transcription factors consists of RelA/p65, c‐Rel, RelB, NF‐κB1 (p50), and NF‐κB2(p52) and are kept silenced in the cytoplasm.^11^ When there are stimuli, the NF‐kappa B complex degrades and p65 enters the nucleus to regulate their target genes.^19^, ^20^ However, direct evidence for the crosslink between CD147 and the NF‐kappa B pathway in HNSCC has been lacking.

In this study, we firstly investigated the expression patterns of CD147 and its clinicopathologic significance in HNSCC. And the role of CD147 in proliferation and invasion of tumor cells were measured both in vivo and in vitro, then the potential relationship with NF‐kappa B pathway was clarified. Taken together, we demonstrated that CD147 promoted tumor progression and metastasis in HNSCC and might be a potential prognostic marker and future treatment target for HNSCC.

## 2 MATERIALS AND METHODS

### 2.1 HNSCC patients and tissue samples

A total of 189 HNSCC patients who received surgery at the Department of Oral and Maxillofacial‐Head and Neck Oncology, Ninth People's Hospital were used in this study. Tissue microarray included 101 tissues of primary HNSCC patients who were diagnosed with oropharyngeal squamous cell carcinoma and 10 normal tissues from outpatient. Additional 88 pairs of HNSCC specimens (HNSCC and non‐tumor adjacent tissues) between 2007 and 2009 were used for quantitative real‐time polymerase chain reaction (qRT‐PCR) and 12 pairs were used for western blot analysis. And the patients who accepted radiotherapy, chemotherapy and/or medicinal treatment were ruled out. The experiments were approved by the Clinical Research Ethics Committee of Ninth People's Hospital, Shanghai Jiao Tong University School of Medicine. All patients wrote the informed consent, in accordance with the provisions of the Helsinki Declaration of 1975.

### 2.2 Cell culture

The human HNSCC cell lines HN4, HN6, HN30, SCC‐9, SCC‐25 and CAL 27 were obtained from the National Institutes of Health. Normal mucosa cells of gingival tissues were taken from patients with impacted teeth extraction. The Rca‐T cell line was purified from tongue squamous cell carcinoma tissues induced by adding 4‐nitroquinoline‐1‐oxide into Sprague‐Dawley rats’ drinking water. HN4, HN6, HN30, CAL 27 and Rca‐T cells were cultured in Dulbecco's modified Eagle's medium (DMEM; GIBCO, USA), containing 10% fetal bovine serum (FBS; GIBCO), 100 units/mL penicillin and 100 μg/mL streptomycin at 37°C in a humidified 5% CO_2_ atmosphere. SCC‐9 and SCC‐25 cells was cultured in DMEM/F12 media (GIBCO), containing 10% FBS (GIBCO), 100 units/mL penicillin and 100 μg/mL streptomycin at 37°C in a humidified 5% CO_2_ atmosphere. Normal mucosa cells were cultured in Defined Keratinocyte‐SFM with Growth Supplement (K‐SFM; GIBCO) at 37°C in a humidified 5% CO_2_ atmosphere.

### 2.3 Immunohistochemistry

Tissues slides were deparaffinized, rehydrated, and then were heated with citric acid buffer for antigenic retrieval. After cooled at room temperature, the sections were submerged into 0.3% hydrogen peroxide for 15 minutes to block endogenous peroxidase activity. After washed in phosphate‐buffered saline (PBS) for 5 minutes, sections were blocked with 5% bovine serum albumin at room temperature for 1 hour. The tissues were incubated with indicated primary antibodies in a humidified chamber at 4°C overnight. After several washes, horseradish peroxidase (HRP)‐labeled goat antimouse or goat anti‐rabbit secondary antibody (Gene Tech, China) was incubated for 50 minutes at room temperature. Hematoxylin and dehydration were used to counterstain the nuclear and slides were dehydrated, and covered with coverslips. Primary antibodies were used at the following dilutions: rabbit CD147 antibody (1:200; CST, USA), rabbit Ki67 antibody (1:200; Abcam, UK).

A tissue microarray containing 101 HNSCC specimens and 10 normal tissues at Shanghai Ninth People's Hospital were used in this study. The immunohistochemistry (IHC) stain score is the sum of positive cell score and staining intensity score for each sample. The positive cell score were classified into five categories: 0%‐25%, scored 1; >25%‐50%, scored 2; >50%‐75%, scored 3; >75%, scored 4. The staining intensity score was defined as 0, 1, 2, and 3. Finally, the above two scores were multiplied for an overall score (ranging from 1 to 12). A total score of 1‐6 was considered low expression; 7‐12 was considered high expression.

### 2.4 Immunofluorescence

Cells were fixed with 4% paraformaldehyde for 15 minutes and permeabilized with 0.2% TritonX‐100 for 10 minutes at room temperature. After washed three times with PBS, cells were incubated with indicated rabbit Phospho‐p65 antibody (1:200; CST) at 4°C overnight. Then, cells were washed and incubated with secondary antibody (HRP rabbit, #8114; CST) and stained with Fluorophore‐conjugated TSA^®^ Plus amplifcation reagent (NEL760001KT), then covered with coverslips using ProLong^®^ Gold Antifade Reagent with DAPI (#8961; CST).

### 2.5 Real‐time PCR

Total RNA was extracted with Trizol Reagent (Invitrogen) following the manufacturer's instructions. The cDNA was synthesized and amplified using the SYBR Premix Ex Taq reagent kit (Takara, Japan) according to the standard protocol. Specific primers for PCR were the following: CD147 forward primers: GAAGTCGTCAGAACACATCAACG, CD147 reverse primers: TTCCGGCGCTTCTCGTAGA; GAPDH forward primers: ACAACTTTGGTATCGTGGAAGG, GAPDH reverse primers: GCCATCACGCCACAGTTTC.

### 2.6 Western blot analysis

Cells or tissues were harvested in RIPA lysis buffer (Yeasen, China) and the concentrations were analysed using the Pierce BCA Protein Assay kit (Thermo Scientific, USA). The cell lysate was electrophoresed by sulfate‐polyacrylamide gel electrophoresis (SDS‐PAGE), transferred to a polyvinylidene fluoride membrane, blocked with non‐fat milk for 1 hour at room temperature and incubated with primary antibodies overnight at 4°C: rabbit CD147 antibody (1:1000; CST), rabbit IKKα antibody (1:1000; CST), rabbit Phospho‐IKKα antibody (Ser176/180) (1:1000; CST), mouse IκBα antibody (1:1000; CST), rabbit Phospho‐IκBα antibody (Ser32) (1:1000; CST), rabbit NF‐kappa B p65 antibody (1:1000; CST), rabbit Phospho‐NF‐kappa B p65 antibody (Ser536) (1:1000; CST), rabbit alpha‐Tubulin antibody (1:1000; Proteintech, USA). Then the membrane was washed three times, incubated with rabbit or mouse HRP‐linked secondary antibodies (1:10000; CST) and visualized with ECL Ultra (New Cell and Molecular Biotech, Suzhou, China).

### 2.7 Lentivirus transfection

We silenced three sequences of the CD147 gene, namely, CD147‐Homo‐550 (siRNA), CD147‐Homo‐647 (siRNA) and CD147‐Homo‐702 (siRNA). We checked the efficiency of the three interference sequences and selected the interference of CD147‐Homo‐550 (siRNA), then packaged it as lentivirus to use in this study (Figure S2C). HN4, HN6 and HN30 cells were transfected with shCD147 lentivirus or control lentivirus (Genomeditech, Shanghai, China) according to the manufacturer's instruction. And the indicated cells were treated with 5 μg/mL puromycin (MedChem Express, China) for 2 weeks to establish stable cell lines. The three interference senquence of CD147 (siRNA) was as follow: CD147‐Homo‐550 (siRNA) sence: CCAGAGUGAAGGCUGUGAATT; CD147‐Homo‐550 (siRNA) antisence: UUCACAGCCUUCACUCUGGTT; CD147‐Homo‐647 (siRNA) sence: GGCCUGGUACAAGAUCACUTT; CD147‐Homo‐647 (siRNA) antisence: AGUGAUCUUGUACCAGGCCTT; CD147‐Homo‐702 (siRNA) sence: AGCAGGUUCUUCGUGAGUUTT; CD147‐Homo‐702 (siRNA) antisence: AACUCACGAAGAACCUGCUTT.

### 2.8 Cell proliferation assay

Cells transfected with shCD147 or shNC were plated at a density of 1 × 10^3^ cells per well in 96‐well plates. CCK‐8 kit (Dojindo, Japan) was used to determine the OD value at 450 nm after 2 hours following the kit instructions. All experiments were performed in triplicate, and the mean proliferation rate was reported.

### 2.9 Colony formation assay

Cells transfected with shCD147 or shNC were plated at a density of 1 × 10^3^ cells per well in 6‐well plates. After 2 weeks, cells were washed with PBS, fixed in 4% paraformaldehyde for 15 minutes, stained with 0.5% crystal violet for 1 hour and were counted at least three times.

### 2.10 Transwell assay

For cell migration assay, 5 × 10^4^ cells transfected with shCD147 or shNC in 200 μL fresh DMEM were plated into upper chamber (Merck Millipore, USA) of the 24‐well plates with 600 μL of DMEM containing 10% FBS in the bottom chamber. After 24 hours, migrated cells were fixed with paraformaldehyde and stained with 10% crystal violet for 1 hour. For cell invasion assay, 50 μL Matrigel (1:8 diluted; BD Biosciences, USA) was first used to coat the chamber, and cells were cultured in a humidified incubator at 37°C for 24 hours and the invasive cells were then harvested and photographed.

### 2.11 Wound‐healing assay

Cells transfected with shCD147 or shNC were cultured in 6‐well plates in DMEM until 100% confluence. The cells were scraped with a P200 tip, washed with PBS and changed for fresh serum‐free DMEM. Pictures were taken at 0, 12 and 24 hours for at least five non‐overlapping fields.

### 2.12 Cell cycle analysis

Cells transfected with shCD147 or shNC were cultured in serum‐free DMEM overnight and replaced with DMEM containing 10% FBS for 24 hours. The cells were washed twice with cold PBS, fixed in cold 70% ethanol overnight, stained with PI staining buffer for 30 minutes at 4°C in the dark and analysed by flow cytometry.

### 2.13 Flow cytometric analysis

To analyse apoptotic cells, adherent and floating cells were harvested, stained with FITC/Annexin V Apoptosis Detection Kit I (BD Pharmingen, USA) according to the instructions and analysed using flow cytometry (FAC SCaliber; BD Biosciences).

### 2.14 Animal experiments

To evaluate the role of CD147 in tumor progression, 1 × 10^6^ HN6‐shNC or HN6‐shCD147 cells in 100 μL serum‐free DMEM were injected subcutaneously into the left or right flanks of 6 week BALB/C nude mice. The weight of the mice and tumor volume were measured every third day for over 20 days. Mice were killed at day 20 and tumors were isolated and photographed. After submerged with formalin and embedded with paraffin, the tissues were performed H&E and IHC experiments. To detect the role of CD147 in lung metastasis, a total of 2 × 10^6^ Rca‐T cells in 200 μL serum‐free DMEM were injected intravenously into the lateral tail vein of the 6‐week‐old BALB/C nude mice. The animals were killed at 0, 1 day, 4 days, 1 week and 2 weeks and the lung tissues were fixed in neutral‐buffered formalin for further experiments. Animal experiments were conducted in accordance with institutional and national regulations for the use and welfare of laboratory animals. And the experiments were approved by the Animal Care and Use Committee of Ninth People's Hospital, Shanghai Jiao Tong University School of Medicine.

### 2.15 Statistical analyses

The data were compiled using with SPSS 19.0 statistical software. Values are presented as means ± SEM. The results of the real‐time PCR, cell proliferation assays, cell cycle assays, colony formation assays, cell scratching assays, cell migration and invasion assays and cell apoptosis assays were compared using Student's *t* test. Kaplan–Meier survival analyses were used to analyse the relationship between CD147 level and the clinicopathologic features. The staining intensity score at 6 was considered to be median score: 1‐6 was considered low expression and 7‐12 was considered high expression. The Cox proportional hazards model was used for univariate and multivariate analyses. All experiments were performed in triplicate and a *P*‐value <0.05 was considered statistically significant.

## 3 RESULTS

### 3.1 Upregulated CD147 expression was associated with aggressive clinicopathologic features and poor prognosis in HNSCC

**Figure 1:**
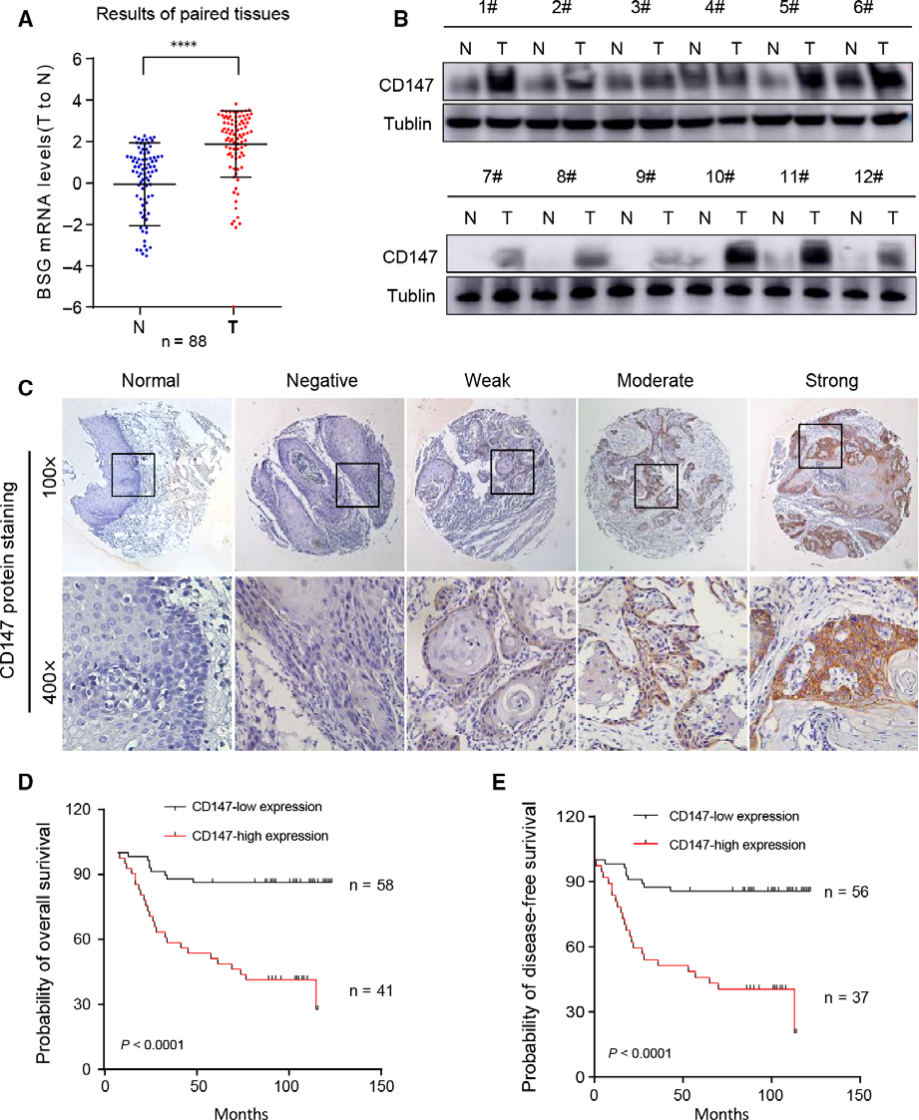
Upregulated CD147 expression was associated with aggressive clinicopathologic features and poor prognosis in HNSCC. (A) The relative CD147 mRNA levels of 88 paired tumor and adjacent non‐tumor tissues in HNSCC is shown. ****P < 0.0001, based on Student's *t* test. (B) The CD147 protein expression levels in 12 paired tumor and adjacent non‐tumor tissues in HNSCC is shown. Tublin was used as a loading control. (C) Immunohistochemistry (IHC) of CD147 expression level on tissue microarrays containing 101 paired HNSCC and 10 normal tissues is shown. Relative negative, weak, moderate and strong PTK7 stain images compared with normal tissues are shown. (D) The probability of overall survival for CD147‐low expression group and CD147‐high expression group of the 101 patients were significantly different (*P* < 0.05). (E) The probability of disease free survival for CD147‐low expression group and CD147‐high expression group of the 101 patients were significantly different (*P* < 0.05). The life status of some patients are missing. Therefore, the total patients are not 101

To detect the role of CD147 in HNSCC, we searched for The Cancer Genome Atlas (TCGA) dataset, and data showed that CD147 expression level was higher in various type of tumors (Figure S1A) and most patients in different tumors was with higher CD147 expression level (Figure S1C). In addition, the CD147 gene was expressed at high levels in HNSCC tissues compared with normal tissues (Figure S1B and D). TCGA dataset also showed that high CD147 expression had a significantly low disease‐free survival (DFS) probability (*P* = 0.026) in HNSCC, however, CD147 expression had no obvious difference with the OS probability (*P* = 0.21) in HNSCC (Figure S1E and F). We further analysed 88 paired HNSCC and non‐tumor adjacent tissues by real‐time PCR and the results demonstrated that the CD147 mRNA expression significantly up‐regulated in HNSCC tumor tissues (*P* < 0.0001) (Figure 1A). Correspondingly, western blot showed that the protein levels of CD147 were also significantly upregulated in the HNSCC tissues compared to the non‐tumor adjacent tissues (Figure 1B). The relationship between variable CD147 expression levels and clinicopathologic features was detected by IHC with a tissue microarray containing 101 HNSCC specimens and 10 normal tissues at the Shanghai Ninth People's Hospital. The results showed relative negative, weak, moderate and strong CD147 staining images from HNSCC patients compared with normal tissues (Figure 1C). As shown in Table 1, CD147 expression levels were significantly associated with gender, nodal status, differentiation and prognosis of HNSCC, but was not significantly associated with age, smoking, drinking and tumor size. Kaplan–Meier was used to analysed the OS probability and DFS probability of HNSCC patients and the results revealed that patients with high CD147 expression had a significantly low OS probability and low DFS probability (both *P* < 0.0001) (Figure 1D and E). The COX regression analyses revealed that CD147 expression was significantly correlated with poor OS in HNSCC patients and was an independent predictor of prognosis for patients with HNSCC (Table 2).

**Table 1:** Association between CD147 expression and clinicopathologic parameters

Variable	N	n (%)	χ^2^	*P*
Age				
>60	37	36.63	4.360	0.113
>45‐≤60	37	36.63		
≤45	27	26.73		
Gender				
Male	53	52.48	4.473	**0.034**
Female	48	47.52		
Site				
Tongue	77	76.24	0.227	0.893
Buccal	12	11.88		
Other	12	11.88		
Smoking				
No	52	51.49	1.442	0.486
Yes	32	31.68		
Unknown	17	16.83		
Alcohol				
No	54	53.47	0.495	0.781
Yes	19	18.81		
Unknown	28	27.72		
Nodal status				
N0	71	70.30	8.642	**0.013**
N1‐2	25	24.75		
Unknown	5	4.950		
Histological grade (differentiation)				
I	37	36.63	15.329	**0.000**
I‐II, II	52	51.49		
II‐III, III	12	11.88		
Tumor size				
T1, T2	46	83.64	4.014	0.260
T3, T4	55	54.46		
Prognosis				
Die	33	32.67	35.060	**0.000**
Survive	66	65.35		
Unknown	2	1.98		

*P*‐values in bold print indicate statistical significance.

**Table 2:** Univariate and multivariate Cox Regression Models for estimating the overall survival

Characteristics	HR	95% CI	*P*‐value
*Univariate analysis*			
Overall survival			
Age (≥60 y vs <60 y)	1.741	0.510‐1.645	0.768
Gender (male vs female)	1.365	0.339‐1.217	0.642
Alcohol history (alcohol vs none alcohol)	2.725	0.419‐1.538	0.508
Smoking history (smoker vs nonsmoker)	1.976	0.667‐3.070	0.357
Histological grade (differentiation)	1.890	0.181‐1.949	**0.037**
Tumor size (T3, T4 vs T1, T2)	1.740	0.400‐1.370	0.339
Nodal status (N1, N2 vs N0)	2.086	0.986‐3.962	**0.021**
CD147 expression (high vs low)	4.030	1.176‐7.425	**0.001**
*Multivariate analysis*			
Overall survival			
Histological grade (differentiation)	1.167	1.023‐3.218	**0.042**
Nodal status (N1, N2 vs N0)	1.814	0.619‐2.197	**0.032**
CD147 expression (high vs low)	3.615	1.052‐5.260	**0.002**

HR, hazard ration; CI, confidence interval.

*P*‐values in bold print indicate statistical significance.

### 3.2 CD147 promoted the proliferation and reduced the apoptosis phenotype of HNSCC cells

**Figure 2:**
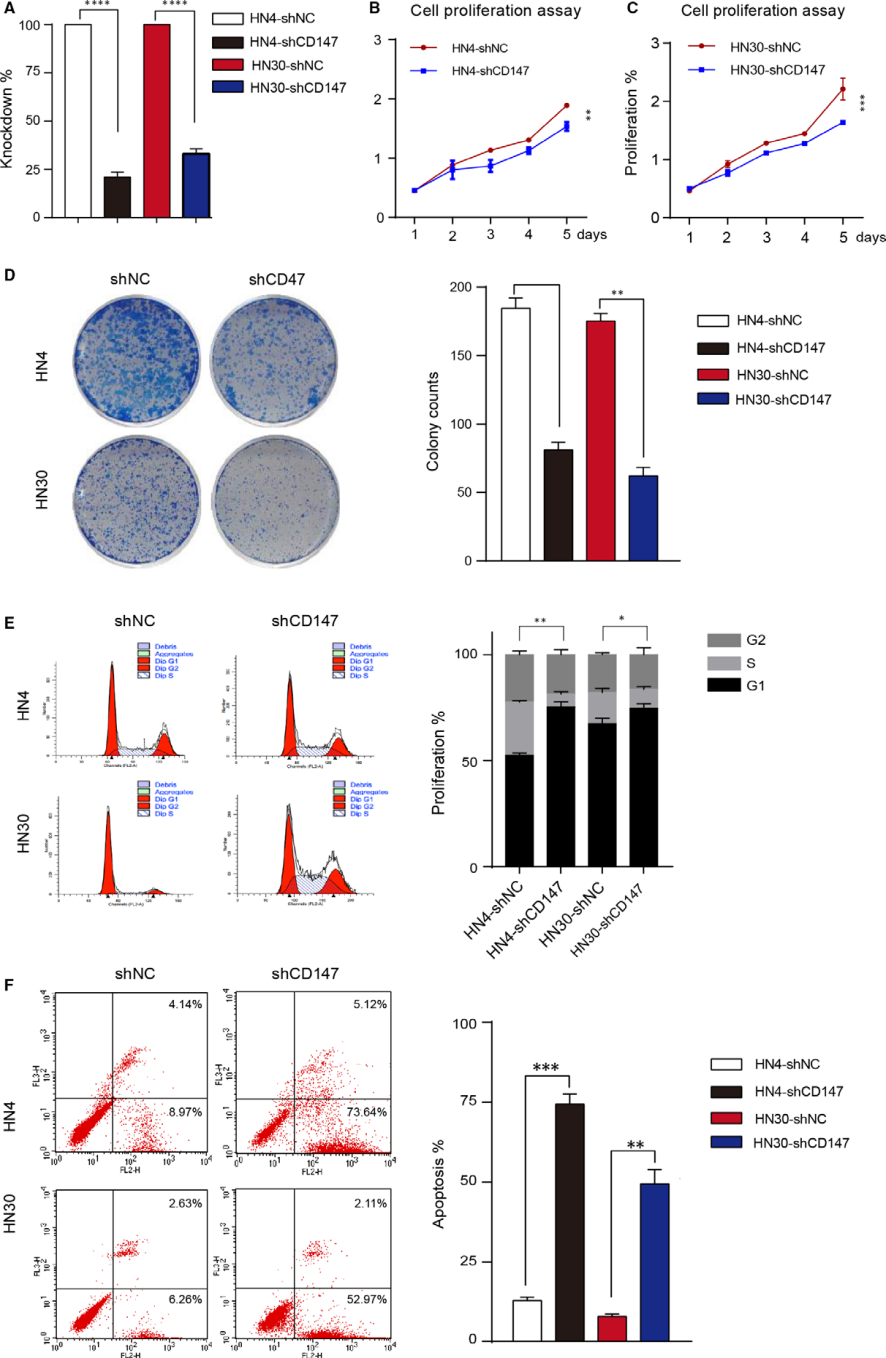
CD147 promoted the proliferation and reduced the apoptosis phenotype of HNSCC cells. (A) The knockdown rate of CD147 in HN4 and HN30 is shown. (B) Proliferation assays showed that proliferation was reduced in HN4 cells after transfected with shCD147 and negative control lentivirus. **P < 0.01, based on Student's *t* test. (C) Proliferation assays showed that proliferation was reduced in HN30 cells after transfected with shCD147 and negative control lentivirus. ***P < 0.001, based on Student's *t* test. (D) The negative control and shCD147 of HN4 and HN30 cells were performed by colony formation assay. **P < 0.01, ***P < 0.001, based on Student's *t* test. (E) Flow cytometry analysis of cell cycle progression in HN4 and HN30 cells were determined after transfected with shCD147 and negative control lentivirus. *P < 0.05, **P < 0.01, based on Student's *t* test. (F) Flow cytometry analysis of apoptosis in HN4 and HN30 cells were performed after transfected with shCD147 and negative control lentivirus. **P < 0.01, ***P < 0.001, based on Student's *t* test

To explore the role of CD147 in proliferation of HNSCC, we first analysed protein expression level of two normal mucosa cells and seven HNSCC cells. The results showed that relatively moderate to high CD147 expression levels were observed in HNSCC tumor cells, whereas low levels were observed in normal mucosa cells (Figure S2A). We then knocked down CD147 in HN4 and HN30 cells with GFP‐tagged lentivirus. Western blot showed that endogenous CD147 expression was efficiently suppressed compared with negative control cells (Figure 2A and Figure S2B). CD147 knockdown significantly reduced proliferation rate of HN4 (*P* < 0.01) and HN30 cells (*P* < 0.001) (Figure 2B and C). In addition, colony formation capacities of HN4 (*P* < 0.001) and HN30 (*P* < 0.01) cells were significantly reduced compared with negative control cells (Figure 2D). Cell cycle arrest and apoptotic cells in HN4 and HN30 transfected with shNC and shCD147 were all consistent with these findings (Figure 2E and F). Taken together, we demonstrated that CD147 promoted proliferation and reduced apoptosis of HNSCC cells.

### 3.3 CD147 promoted the migration and invasion phenotype of HNSCC cells

**Figure 3:**
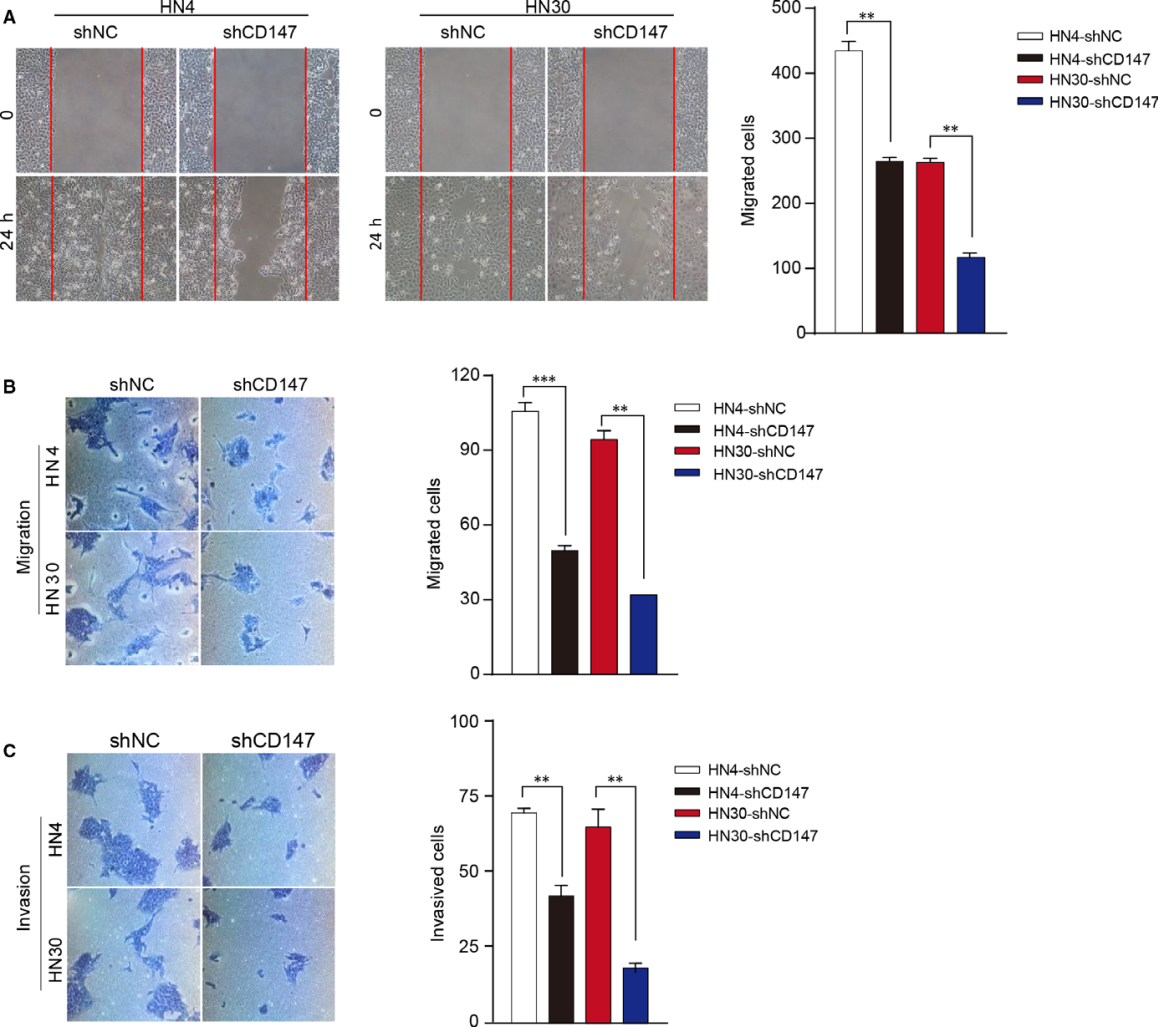
CD147 promoted the migration and invasion phenotype of HNSCC cells. (A) HN4 and HN30 cells were transfected with shCD147 and negative control lentivirus, subsequently analysed with scratch wound healing assay. **P < 0.01, based on Student's *t* test. (B) HN4 and HN30 cells were transfected with shCD147 and negative control lentivirus, subsequently analysed with migration assays. **P < 0.01, ***P < 0.001, based on Student's *t* test. (C) HN4 and HN30 cells were transfected with shCD147 and negative control lentivirus, subsequently analysed with invasion assays. **P < 0.01, based on Student's *t* test

Based on the role of CD147 in the proliferation of HNSCC cells, we further detected the effect of CD147 on tumor migration and invasion. Scratch wound healing assays showed that CD147 knockdown significantly reduce the migration of HN4 and HN30 cells (both *P* < 0.01) (Figure 3A). In addition, migration and invasion assays were also associated with the results (Figure 3B and C). Collectively, these data showed that CD147 promoted the migration and invasion phenotype of HNSCC cells.

### 3.4 Knockdown of CD147 in HNSCC cells reduced tumor growth in vivo

**Figure 4:**
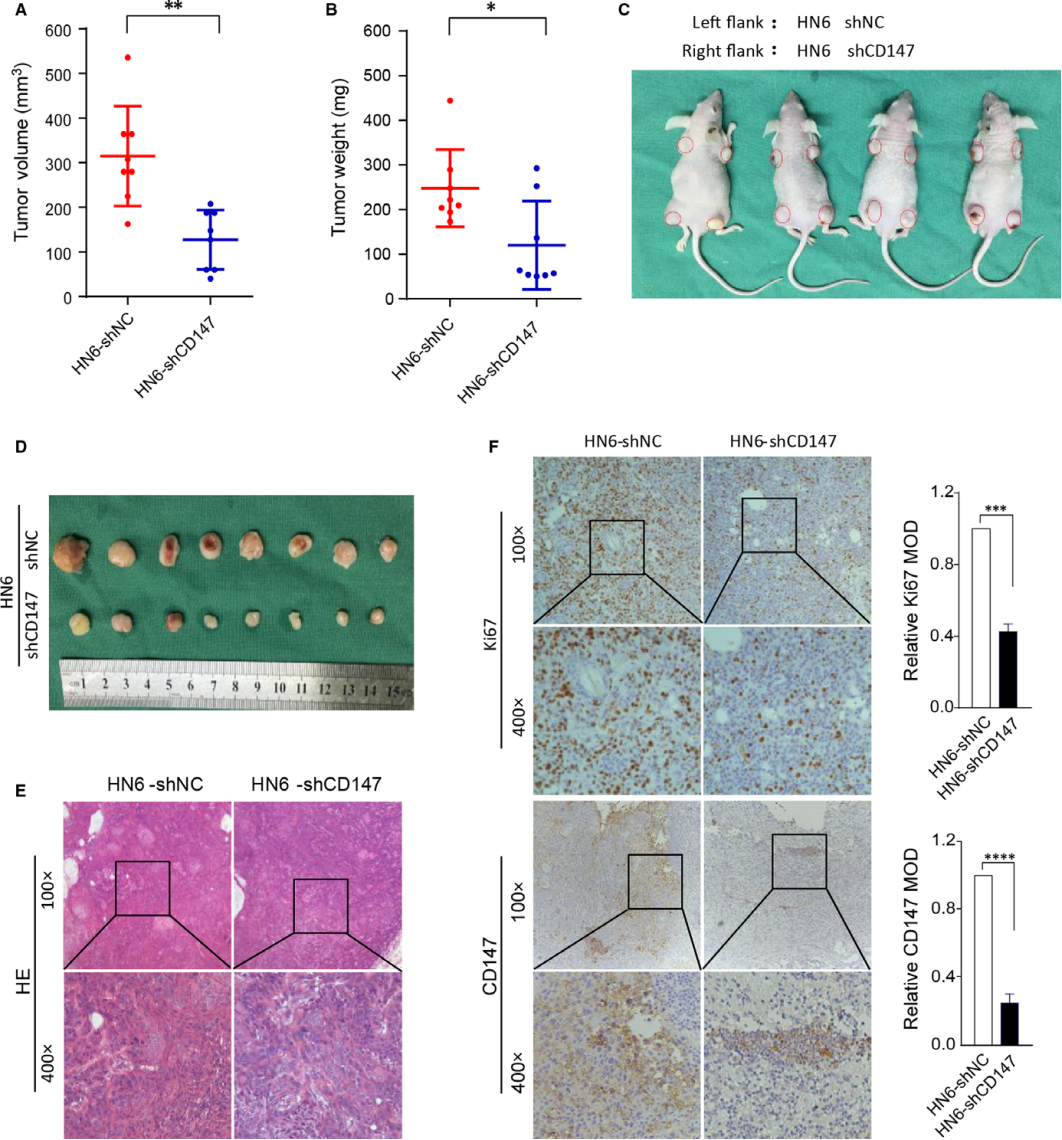
Knockdown of CD147 in HNSCC cells reduced tumor growth in vivo. (A) A total of 1 × 10^6^ HN6 negative control cells and shCD147 cells in 100 μL fresh DMEM medium were subcutaneously injected into the left or right flank of the BALB/C nude mice. Tumor volume of two groups are presented. **P < 0.01, based on Student's *t* test. (B) Tumor weight of two groups are presented. *P < 0.05, based on Student's *t* test. (C) Tumor growth 20 days after injection of the BALB/C nude mice are shown. (D) Mice were killed and the tumors are isolated. (E) Representative H&E staining of tumor tissues is shown. (F) Immunohistochemical staining of Ki67 and CD147 in shNC and shCD147 xenografted tumors is shown. ***P < 0.001, ****P < 0.0001, based on Student's *t* test

To examine the effect of CD147 on tumorigenicity in vivo, we established a xenograft model in BALB/C nude mice. A total of 1 × 10^6^ HN6 negative control cells and shCD147 cells in 100 μL fresh DMEM medium were subcutaneously injected into the left or right flank of the BALB/C nude mice. Tumor growth was recorded for overall 20 days and mice were killed (Figure 4C). The results showed that the tumor volume and weight of HN6 shCD147 cells were significantly inhibited than HN6 negative control cells (*P* < 0.05) (Figure 4A, B and D). There were no morphological difference between HN6 negative control group and HN6‐shCD147 group in xenografted tumors (Figure 4E). Immunohistochemistry assays showed that CD147 knockdown reduced the expression of Ki67 in xenograft tumors (*P* < 0.0001) (Figure 4F). Consistent with the data in vitro, these studies demonstrated that knockdown of CD147 in HNSCC cells reduced tumor growth in vivo.

### 3.5 CD147 expression was higher in lung metastasis tissues in vivo

**Figure 5:**
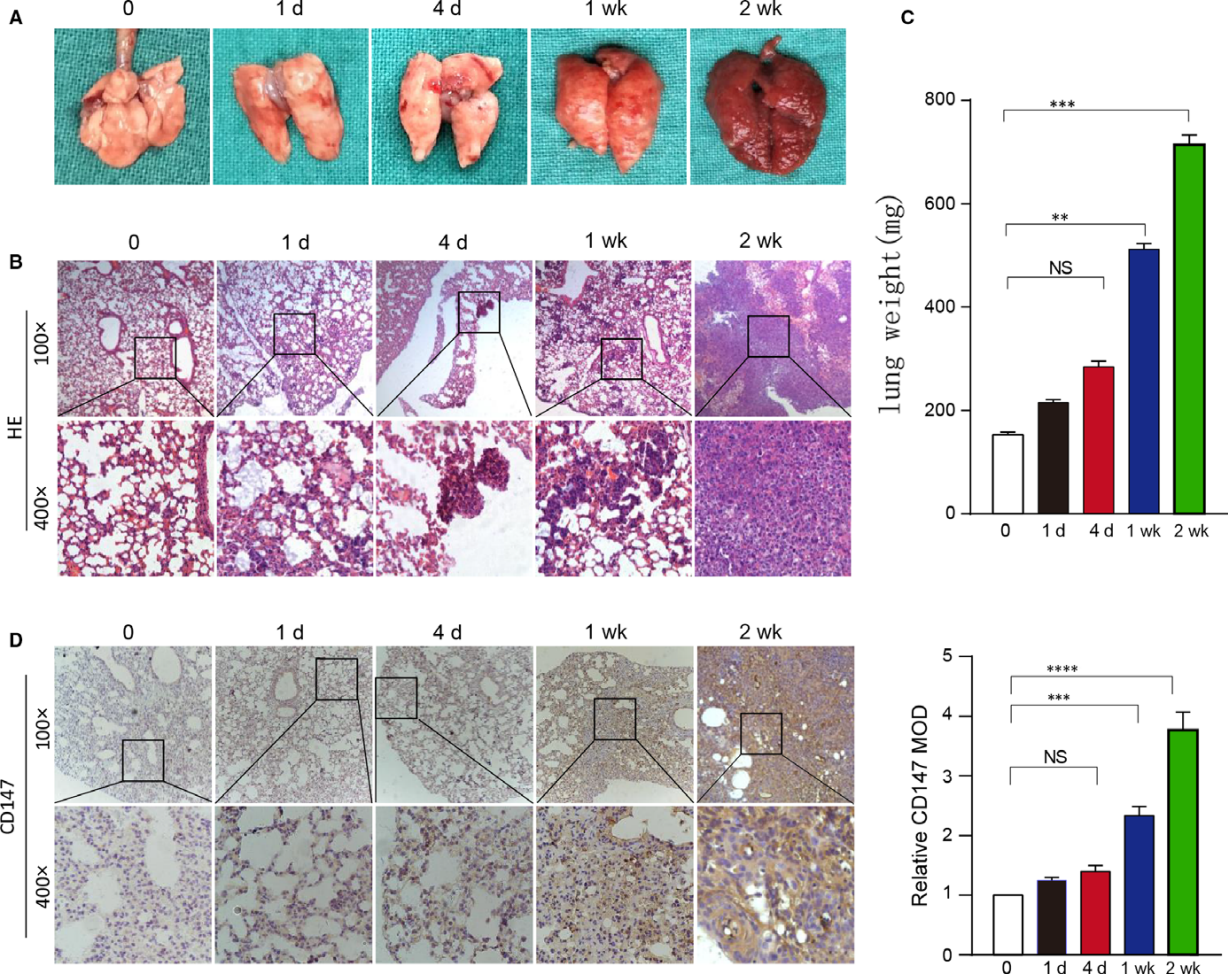
CD147 expression was higher lung metastasis tissues in vivo. (A) Whole mount pictures of the lung tissues are shown. (B) H&E staining of lung tissue sections at 0, 1 day, 4 days, 1 week and 2 weeks is shown. (C) The lung weight of BALB/C nude mice killed at 0, 1 day, 4 days, 1 week and 2 weeks are shown. ****P < 0.0001, based on Student's *t* test. (D) Immunohistochemical staining of CD147 in lung tissue sections at 0, 1 day, 4 days, 1 week and 2 weeks is shown. ****P < 0.0001, based on Student's *t* test

To investigate the role of CD147 in lung metastasis, a total of 2 × 10^6^ Rca‐T cells were injected into the lateral tail vein of nude mice and mice were killed at 0, 1 day, 4 days, 1 week and 2 weeks respectively. Then the lungs were isolated and pictures were taken (Figure 5A). H&E staining revealed that the metastatic colony numbers in lungs at 1 week and 2 weeks were significantly higher compared to control groups (Figure 5B). Lung weight were also significantly elevated at 1 week (*P* < 0.01) and 2 weeks (*P* < 0.001), but not significantly elevated at 1 day and 4 days compared with 0 day (Figure 5C). We further explored the CD147 expression level at different points, and the results demonstrated that CD147 expression levels were higher in lungs of 1 week (*P* < 0.001) and 2 weeks (*P* < 0.0001) compared with 0 day (Figure 5D). Therefore, we proposed that CD147 may take part in the metastatic clones formation in vivo.

### 3.6 CD147 activated NF‐kappa B signaling in HNSCC

**Figure 6:**
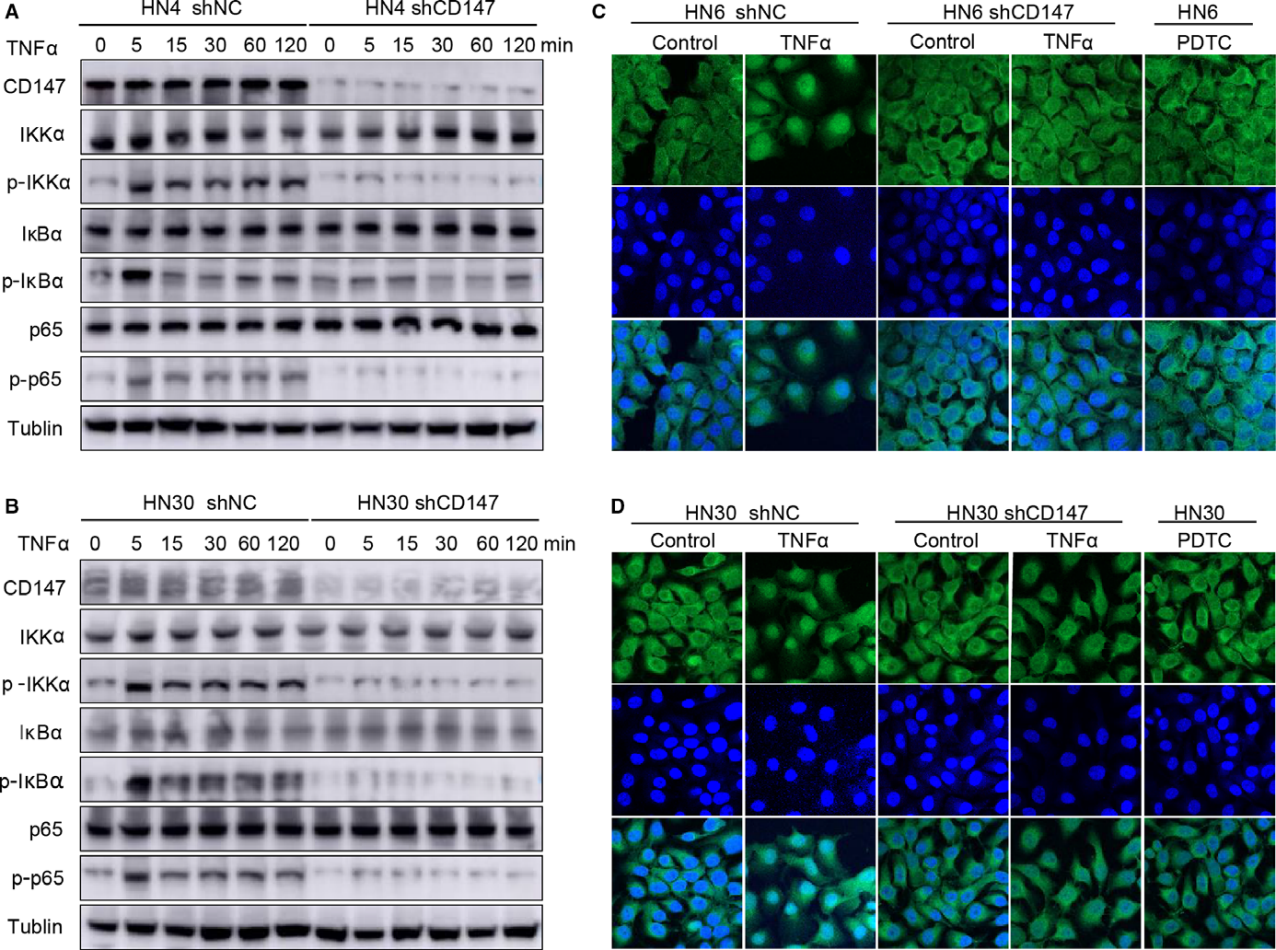
CD147 activated NF‐kappa B signaling in HNSCC. (A) After being starved with serum‐free DMEM overnight, HN4 shCD147 and negative control cells were treated with 5 ng/mL TNFα for 0, 5, 15, 30, 60, 120 min. Lysates were prepared, and CD147, IKKα, p‐IKKα, IκBα, p‐IκBα, p65, p‐p65 were analysed by western blotting. Tublin was used as a loading control. (B) After being starved with serum‐free DMEM overnight, HN30 shCD147 and negative control cells were treated with 5 ng/mL TNFα for 0, 5, 15, 30, 60, 120 min. Lysates were prepared, and CD147, IKKα, p‐IKKα, IκBα, p‐IκBα, p65, p‐p65 were analysed by western blotting. Tublin was used as a loading control. (C) After being starved with serum‐free DMEM overnight, HN6 shNC and shPTK7 cells were treated with 5 ng/mL TNFα or serum‐free DMEM for 5 min, and HN6 was treated with PDTC for 5 min, then the location of p65 was detected by laser scanning microscopy. (D) After being starved with serum‐free DMEM overnight, HN30 shNC and shPTK7 cells were treated with 5 ng/mL TNFα or serum‐free DMEM for 5 min, and HN30 was treated with PDTC for 5 min, then the location of p65 was detected by laser scanning microscopy

As studies have shown, CD147 enhanced tumor growth through NF‐kappa B signalling.^21^, ^22^ To observe the time‐dependent activation of NF‐kappa B signaling, negative control cells and shCD147 cells were treated with serum‐free DMEM overnight, subsequently stimulated with TNFα and cells were harvested at 0, 5, 15, 30, 60 and 120 min. Interestingly, the protein expression of p‐IKKα, p‐IκBα and p‐p65 in shNC cells significantly increased from 5 min after stimulated with TNFα, but the protein expression in shCD147 cells had no obvious upregulation in both HN4 and HN30 cells (Figure 6A and B). Consistent with these results, the immunofluorescence assay also demonstrated that when treated with TNFα, p65 of HN4 and HN30 negative control cells entered into the nucleus, but p65 of HN4 and HN30 shCD147 cells was still in the cytoplasm. The HN4 and HN30 cells that were treated with PDTC were used as negative control (Figure 6C and D). Taken together, we concluded that CD147 played a role in HNSCC through activating NF‐kappa B signaling.

## 4 DISCUSSION

Multimodality approaches have improved clinical outcome in HNSCC, including surgery, chemotherapy, radiotherapy and therapeutics targeted EGFR.^23^, ^24^ However, there are still 50% of patients uncured,^4^, ^11^ which suggested an urgent need to detect other predictable biomarkers in HNSCC.

As our previous study shown, CD147 is considered to be a HNSCC cell surface marker that could enhance the tumor initiation and progression.^5^ Studies also demonstrated that CD147 had negative role in regulating in T cell‐mediated immune responses and drugs targeting CD147 had anti‐angiogenesis and anti‐invasion activity.^25^, ^26^, ^27^, ^28^ Our data determined that CD147 exerted positive effect on tumorigenicity and cancer progression of HNSCC both in vitro and in vivo. In addition, CD147 expression level is positively correlated with clinical outcome and tumor histological grade in HNSCC. Hence, CD147 might be predictable diagnosis target for HNSCC.

In addition, reports showed that CD147 is an important molecule that induced NF‐kappa B pathway activated and cause increased cell proliferation and reduced cell apoptosis.^29^ NF‐kappa B signaling also activated recombinant human CD147 in cardiomyocytes.^15^ Therefore, we investigate whether CD147 exerted its function through NF‐kappa B pathway. We focused on NF‐kappa B pathway for three reasons. First, many previous studies demonstrated the molecular link between CD147 and NF‐kappa B pathway.^15^, ^29^, ^30^ Second, NF‐kappa B target several genes that are important for the inflammatory response, cell proliferation, adhesion, interaction with the microenvironment, as well as tumor growth and metastasis.^31^, ^32^, ^33^, ^34^ Third, our previous study demonstrated that NF‐kappa B activity was significantly positively correlated with invasion and metastasis in HNSCC.^35^ Inhibition of NF‐kappa B activity suppressed cell migration in vitro and reduced the lung metastasis and lymph node metastasis in vivo.^35^ In this study, CD147 knockdown was also correlated with reduced proliferation, migration and invasion, increased apoptosis of HNSCC cells, and suppressed tumor progression in vivo, which suggested that the role of CD147 was consistent with NF‐kappa B in HNSCC. In addition, our study demonstrated that CD147 knockdown significantly impaired the TNF‐α induced NF‐kappa B activation. Therefore, we concluded that CD147 promoted tumor initiation and progression of HNSCC via NF‐kappa B signaling.

The mechanisms underlying CD147 induced activation of NF‐kappa B in HNSCC were still not clear. Reports showed that different stimuli could activate the NF‐kappa B signaling, including cytokine, growth factor, DNA damages and oncogenic stress.^11^ The activation of NF‐kappa B induced the formation of the IKK (IκB kinase) protein complex and some protein regulate the complex as IKK interactors, such as Hsp70, Hsp90. And some NF‐kappa B targeted genes promote tumor growth, invasion and metastasis, such as MMP‐9 and IL‐6.^11^, ^30^, ^35^, ^36^ There are reports shown that CD147 promoted the expression of MMP‐9, which might be a mechanism that CD147 promoted tumor progression and invasion in HNSCC via regulating NF‐kappa B signaling.^30^ In addition, inhibition of CD147 by using the CD147 monoclonal antibody (5A12) was reported to reduce the proinflammatory cytokine production and thus suppressed the activation of NF‐kappa B pathway.^29^ Furthermore, in the progress of tumor progression, tumor cells might interact with stimuli from the host environment, including tissue‐ or organ‐specific cytokines.^37^ CD147, a type I transmembrane glycoprotein of the immunoglobulin superfamily, is likely to accept different stimuli from their immediate environment and thus contribute to the stimuli‐induced NF‐kappa B activation, which then promoted tumor progression. Taken together, all these are possible mechanisms for CD147 induced NF‐kappa B pathway activation in HNSCC.

Summarily, in this study, we identified CD147 as a tumor promotor in HNSCC cells and CD147 could act as an independent predictor for prognosis of patients with HNSCC. Furthermore, the loss of CD147 expression on cell surface might be a predictive therapeutic target.^27^ However, there remains some problems that need to be solved. First, whether there are other cell surface markers that could have joint effect with CD147. If so, whether the two or more markers would form a complex that has much significantly effect on tumor progression? Second, we did not clarify the definite mechanism underlying CD147 induced NF‐kappa B activation. It is still unclear whether CD147 accepts the stimuli from environment and then activates the NF‐kappa B signaling or CD147 increases expression of some protein which might interact with NF‐kappa B complex or IKK complex. Third, concrete roles of CD147's extracellular and intracellular portion in HNSCC were still unknown. Therefore, further studies are still necessary to confirm underlying mechanism of CD147 in tumor initiation and progression of HNSCC. In addition, targeted CD147 drug or monoclonal antibody might be effective therapies for HNSCC future treatment.

## CONFLICT OF INTEREST

The authors declare no conflict of interest.

## AUTHOR CONTRIBUTION

MY and WC performed conception and design; BY and YZ contributed to experiments performing and wrote the manuscript; BY and YJ performed data analysis; KW collected the tissue samples information of patients and generated the data; LW analysed the staining and results of pathological section; MY and WC provided financial support and final approval of manuscript.
