# Supplementary figures and images for: CD147 promotes progression of head and neck squamous cell carcinoma via NF‐kappa B signaling
Source: J Cell Mol Med. 2018 Nov 12;23(2):954–66. doi: 10.1111/jcmm.13996 (PMC6349162; doi:10.1111/jcmm.13996)

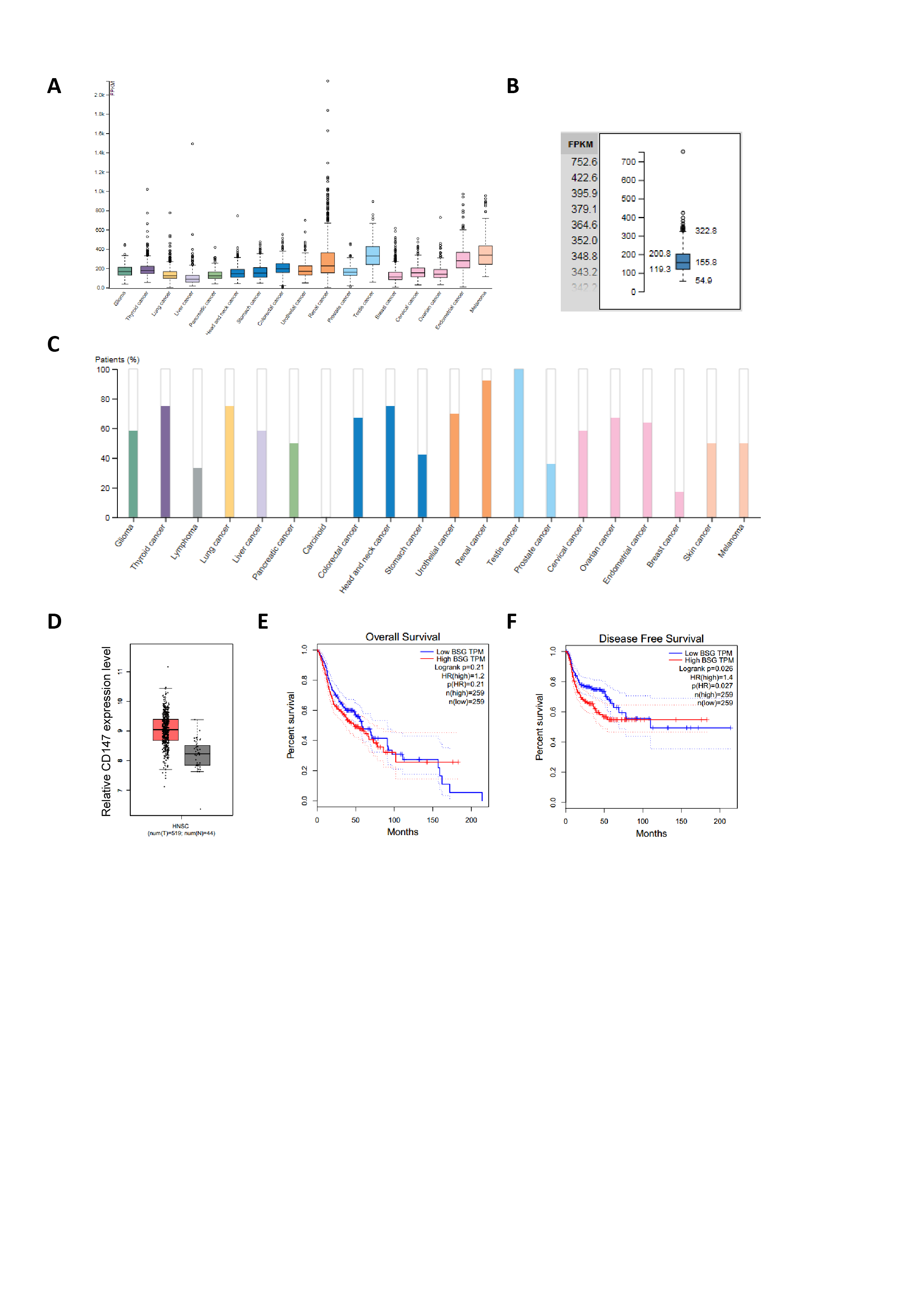

Supplement: Supplementary file 1 [file JCMM-23-954-s001.tif]

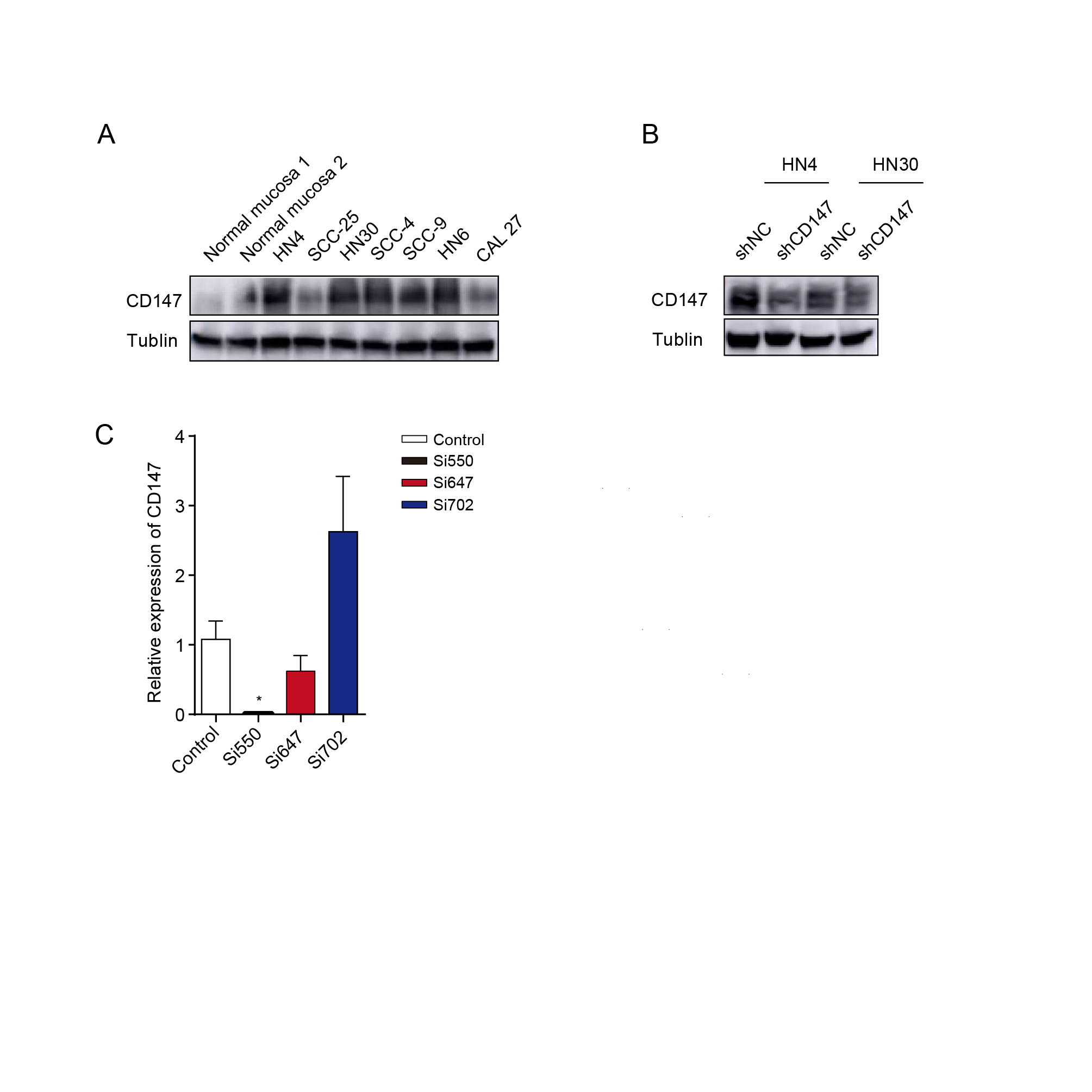

Supplement: Supplementary file 2 [file JCMM-23-954-s002.tif]
