# Supplementary material for: CD147 promotes progression of head and neck squamous cell carcinoma via NF‐kappa B signaling
Source: J Cell Mol Med. 2018 Nov 12;23(2):954–66. doi: 10.1111/jcmm.13996 (PMC6349162; doi:10.1111/jcmm.13996)
Supplement: Supplementary file 3 [file JCMM-23-954-s003.docx]

**Supplementary Figure 1**

(A) Relative CD147 expression levels in many types of tumors from TCGA database are shown.

(B) Relative CD147 expression levels in HNSCC are shown.

(C) The percent of patient with high CD147 mRNA expression levels in many types of tumors from TCGA database are shown.

(D) Relative CD147 mRNA expression levels in HNSCC and adjacent non-tumor tissues from TCGA database were shown.

(E) The percent of Overall Survival for CD147-low expression group and CD147-high expression group was shown (*p*=0.21).

(F) The percent of Disease Free Survival for CD147-low expression group and CD147-high expression group was shown (*p*=0.026).

**Supplementary Figure 2**

(A) The CD147 protein level of HN4 and HN30 cell lines were detected by western blot after stably transfected with shCD147 and negative control lentivirus. Tublin was used as a loading control.
